# Phase 3 Evaluation of an Innovative Simple Molecular Test for the Diagnosis of Malaria and Follow-Up of Treatment Efficacy in Pregnant Women in Sub-Saharan Africa (Preg-Diagmal)

**DOI:** 10.3390/tropicalmed7090219

**Published:** 2022-09-01

**Authors:** Francois Kiemde, Norbert van Dijk, Halidou Tinto, Hypolite Muhindo-Mavoko, Daniel Valia, Berenger Kaboré, Japhet Kabalu, Vivi Maketa, Petra Mens, Henk Schallig

**Affiliations:** 1 Institut de Recherche en Sciences de la Santé—Clinical Research Unit of Nanoro (IRSS-CRUN), Nanoro 18, Burkina Faso; 2 Amsterdam University Medical Centers, Academic Medical Centre at the University of Amsterdam, Department of Medical Microbiology and Infection Prevention, Laboratory for Experimental Parasitology, Meibergdreef 9, 1105 AZ Amsterdam, The Netherlands; 3 Amsterdam Institute for Infections and Immunology, Infectious Diseases Programme, Meibergdreef 9, 1105 AZ Amsterdam, The Netherlands; 4 Department of Tropical Medicine, University of Kinshasa (UNIKIN), Kinshasa 7948, Congo

**Keywords:** malaria, pregnant women, rapid diagnostic test, db-PCR-NALFIA, diagnosis, *Plasmodium*

## Abstract

The malaria parasite *Plasmodium falciparum* (*Pf*) can sequester in the placenta resulting in low density of peripheral parasitemia and consequently in false negative malaria diagnosis (by microscopy) in pregnant women. Moreover, the use of rapid diagnostic tests (RDTs) in diagnostic strategies, including those for the detection of a malaria infection during pregnancy, is constrained by either persistent malaria antigen (histidine-rich protein 2; HRP2) after successful treatment, leading to false positive test results, or by false negative results as previously mentioned due to parasite sequestration (which is further exacerbated due to the low limited of detection [LoD] of conventional RDTs) or to HRP2 deletion. Recently, a direct blood polymerase chain reaction combined with a nucleic acid lateral flow immunoassay (dbPCR-NALFIA) has been developed, which circumvents these challenges and has demonstrated its diagnostic potential in phase 1 and 2 studies. The PREG-DIAGMAL trial presented in this manuscript will assess the diagnostic performance of dbPCR-NALFIA for the diagnostic of malaria in pregnant women and its potential to monitor treatment efficacy in these subjects. The work is ancillary embedded in an ongoing EDCTP funded trial, the PyraPreg project (PACTR202011812241529) in which the safety and efficacy of a newly registered Artemisinin-Based Combination (Pyronaridine-Artesunate) is being evaluated in pregnant women. This is a Phase 3 diagnostic evaluation conducted in 2 African countries: Democratic Republic of the Congo (DRC) and Burkina Faso. Pregnant women fulfilling the inclusion criteria of the PyraPreg study will be also invited to participate in the PREG-DIAGMAL study. Diagnostic accuracy will be assessed following the WHO/TDR guidelines for the evaluation of diagnostics and reported according to STARD principles. Due to the lack of a 100% specific and sensitive standard diagnostic test for malaria, the sensitivity and specificity of the new test will be compared to the available diagnostic practice in place at the selected settings (microscopy and/or RDT) and to quantitative PCR as the reference test. This phase 3 diagnostic study is designed towards the evaluation of the performance of a new diagnostic tool for the screening of malaria and the monitoring of treatment in pregnant women under real conditions life. If successful, the dbPCR-NALFIA could be a valuable tool to add to the diagnostic arsenal for malaria, in particular during pregnancy. Trial registration: Pan African Clinical Trial Registry database (PACTR202203780981413). Registered on 17 March 2022.

## 1. Background

Malaria in humans is a life-threatening disease caused by single-cell (protozoan) parasites of the genus *Plasmodium*: *P. falciparum*, *P. vivax*, *P. ovale*, *P. malariae,* and *P. knowlesi*. Malaria is a major public health problem in (sub-) tropical regions with approximately half of the world’s population being at risk. In 2019, about 229 million clinical malaria cases and nearly 409,000 deaths were reported worldwide. The majority of the cases (94%) occurred in sub-Saharan Africa, with young children and pregnant women being most at risk [1]. Malaria management has been greatly improved due to the wide implementation of World Health Organization (WHO) recommendations that all suspected malaria cases should have a parasite-based diagnosis prior to initiating treatment with artemisinin-based combination therapy (ACT) [2]. Malaria parasite specific antigen-detecting Rapid Diagnostic Tests (RDTs) are considered as appropriate tools to manage febrile illnesses in malaria endemic settings [1]. The short time needed to obtain a diagnostic result, simple read-out, easy performance by non-technical staff, and relatively low costs have contributed to the acceptance of RDTs in differentiating malaria from other causes of fever [3]. Most commonly used RDTs are the *P. falciparum* histidine-rich protein 2 (*Pf*HRP2) based RDT, specific for *P. falciparum* only, and the *Plasmodium* lactate dehydrogenase (*p*LDH)-based RDT that detects all *Plasmodium* species, or combination tests with two test lines (a *P. falciparum*-specific line and an all *Plasmodium*-test line) [4,5].

However, the success of RDTs’ implementation is constrained by persisting *Pf*HRP2 after successful treatment, which may lead to false positive test results [6,7,8]. Furthermore, studies report false positive diagnoses by *Pf*HRP2-based RDT particularly in seasonal malaria transmission settings or under harsh environmental conditions [9,10]. Of greater concern are false negative *Pf*HRP2-RDT results increasingly reported from malaria endemic regions due to *hrp*2 deletions in the *Plasmodium* genome [11,12,13]. In these settings, a HRP2-based RDT will be negative, even if a *P. falciparum* infection is present. Finally, RDTs (*p*LDH-based in particular) have sensitivity limitations, also resulting in false negative results [5,14]. This is particularly evident in the case of pregnant women where the malaria parasites, in particular *P. falciparum¸* sequester in the placenta (placental malaria). Consequently, in pregnant women, peripheral parasitaemia can be absent or below the detection limit of RDTs [15,16].

Although malaria microscopy remains the standard for clinical diagnotis, the limit of detection (LoD) of malaria parasites by expert microscopy (around 10 parasites/μL) could miss low parasitaemia. Furthermore, the LoD depends on the microscopic reading technique, the experience of the reader, and the availability of a well-maintained microscope [17]. 

In this context, there is a pressing need to develop more sensitive and accurate malaria diagnostic tests that circumvent the limitations of RDTs and microscopy. In general, molecular biology-based diagnostic tests are considered to be the best methods to detect malaria infections, irrespective of low parasite densities or HRP2 deletions [18,19,20]. Several molecular methods have been developed, including conventional and quantitative real-time polymerase chain reaction (qPCR), nucleic acid sequence-based amplification, or loop mediated amplification [19]. These platforms are highly sensitive and can allow species differentiation and parasite quantification. These formats would be ideal for patient management, but their relative complexity hampers their use as near point of care (PoC) tests. In particular samples’ processing (DNA extraction), and complicated read-out systems (gel electrophoresis with UV reading or computerised systems) hamper wide implementation [19].

To circumvent these challenges, a simple molecular diagnostic platform to diagnose malaria has been developed that can be used near patient (PoC). This platform combines direct-on-blood (db)PCR with a nucleic acid lateral flow immunoassay (NALFIA) to detect *Plasmodium* species in a multiplex format [21]. The NALFIA is a simple read-out system, comparable to a dipstick, to visualize amplicons. The dbPCR-NALFIA system has an analytic sensitivity of 1.0 to 0.1 parasite/µL of blood (10–100 times more sensitive than conventional malaria diagnostics) with a high sensitivity and specificity in clinical settings [21]. Importantly, the platform allows for direct amplification of *Plasmodium* DNA in whole blood [22] by utilizing specific enzymes and buffers that are able to withstand the inhibitory effects usually encountered when directly amplifying DNA in blood. This avoids contamination-prone and labour-intensive parasite DNA extraction from a clinical sample. This dbPCR-NALFIA has passed laboratory evaluations and trials in disease endemic countries [22,23]. Recently the dbPCR-NALFIA has been optimised by using a mini-PCR system (battery operated, reducing its dependency on electricity, solar powered and controlled via an application on a mobile telephone) thereby enhancing its field applicability. Currently, the platform (mini-dbPCR-NALFIA) is undergoing phase III diagnostic evaluations in the framework of the European & Developing Countries Clinical Trials Partnership (EDCTP) funded DIAGMAL project (PACTR202202766889963 and ISRCTN13334317) in 5 African countries: Ethiopia, Burkina Faso, Namibia, Sudan, and Kenya. In this project, the diagnostic performance of the mini-dbPCR-NALFIA test will be evaluated in the context of different malaria epidemiology settings. Furthermore, a cost effectiveness evaluation of the implementation of this new diagnostic platform will be performed in the framework of this study.

These above-mentioned evaluations do not include pregnant women who are however very vulnerable to malaria infections [24,25]. Indeed, *P. falciparum* can sequester in the placenta during pregnancy, resulting in low peripheral parasitemia. Consequently, malaria diagnostic tests are often false negative in pregnant women who actually do have a *P. falciparum* malaria infection [26,27] and they do not receive adequate treatment. Due to its very high analytical sensitivity, the mini-dbPCR-NALFIA test could be a good diagnostic test to properly detect malaria in pregnancy (MiP). 

In addition, the treatment of malaria in pregnancy needs to be safe and efficient. Currently most cases of MiP are treated with ACT [24,28]. It is however difficult to measure the effect of treatment in these specific cases. The mini-dbPCR-NALFIA might provide a suitable platform to follow treatment efficacy as well.

The main aim of the study is to evaluate the diagnostic performance of the mini-dbPCR-NALFIA test for MiP. In order to achieve that, the following study objectives will be achieved through a dedicated diagnostic trial focussing on malaria infection in pregnant women.

The planned work has the following primary objective: to assess the diagnostic accuracy of the mini-dbPCR-NALFIA test for the diagnosis of malaria in pregnancy compared to routine diagnostic procedure(s) in place (microscopy and/or RDTs) and qPCR (as reference test). The secondary objectives of the trial are:

To determine the utility of the mini-dbPCR-NALFIA to monitor anti-malaria treatment with ACT efficacy in pregnancy during a follow-up period of 63 days.

## 2. Design/Methods

The trial design is presented below by following the SPIRIT guidelines. This trial is an ancillary study embedded in an EDCTP funded drug trial (Safety and Efficacy of a newly registered Artemisinin-Based Combination (Pyronaridine-Artesunate-PYRAMAX^®^) (PyraPreg [PACTR202011812241529]). The planned phase 3 diagnostic study consists of two components.

The first component will assess the diagnostic accuracy of the mini-dbPCR-NALFIA test for the diagnosis of malaria in pregnant women (**diagnostic screening part**). The study hypothesis for this component is: “mini-dbPCR-NALFIA will offer an improved diagnostic platform over microscopy and/or currently available RDTs for the detection of malaria infection in pregnant women”. This will be studied through a phase 3 diagnostic trial. Diagnostic performance will be assessed following the WHO/TDR guidelines for the evaluation of diagnostics [29] and the results will be reported according to STARD principles [30]. This phase 3 evaluation will screen pregnant women at the point of enrolment for the main PyraPreg trial for the presence of malaria in both Burkina Faso and DRC. Potential study participants (≥15 years of age, pregnant, able to provide informed consent) will be identified during their visits to the study health facility and asked to participate in the diagnostic study. Consequently, both malaria negative, as well as malaria positive (confirmed by the standard diagnostic practices in place), will be tested for this screening part of the study. An additional blood sample (finger prick blood), next to the clinical sampling performed in the framework of the PyraPreg project at the point of screening, will be collected from the same women. This sample will be analysed by mini-dbPCR-NALFIA (index test) and qPCR (as reference test). The results of the experimental diagnostic testing will not be used to make any decisions about the clinical management of pregnant women.

For the **treatment follow-up part** (second component), we will recruit the same women who have been recruited for the main PyraPreg trial (in Burkina Faso only) and who will be followed up for a period of 63 days. At each of their planned study visit (at days 1, 2, 3, 7, 14, 21, 28, 35, 42, 49-56, and 63) we will obtain an additional blood sample for qPCR and mini-dbPCR-NALFIA testing.

### 2.1. Methods: Participants, Test evaluation and Outcomes

#### 2.1.1. Study Sites

The study will be carried out in 2 African countries (Burkina Faso and DRC).

In Burkina Faso, the study will be conducted by the Clinical Research Unit of Nanoro (CRUN), located in central—west Burkina Faso, approximately 90 Km from the capital city, Ouagadougou. Malaria is the main public health problem and represents a significant burden, particularly for pregnant women and children. The prevalence of malaria in pregnancy is estimated to be around 25% in the region.

In DRC, the study will be conducted by the Department of Tropical Medicine, UNIKIN. Participants will be recruited in the Kinshasa suburb, where malaria transmission is perennial and the prevalence is estimated at around 30%. 

#### 2.1.2. Eligibility Criteria

The screening study will include cases who fulfil the following inclusion criteria:

1. Gestation ≥16 weeks and <37 weeks as assessed by ultrasound (if possible) or uterine height or late menstrual period;
2. Age: ≥15 years;
3. Residence within the health facility catchment area;
4. Ability to provide written informed consent.

The treatment follow-up study will include cases that have met the inclusion criteria for the PyraPreg study. These have been defined as follows:

1. Gestation ≥16 weeks and <37 weeks as assessed by ultrasound (if possible,) or uterine height or late menstrual period;
2. *P. falciparum* mono-infection (by microscopy) of any density, regardless of symptoms and HIV status;
3. Haemoglobin ≥7 g/dL;
4. Age: ≥15 years;
5. Residence within the health facility catchment area;
6. Willing to adhere to study requirements and to deliver at the health facility;
7. Ability to provide written informed consent.

Not meeting the inclusion criteria will result in exclusion from the study.

#### 2.1.3. Who will Take Informed Consent?

Written informed consent will be obtained from all the study participants before enrolment. After an interview with the study physician or a member of the study research team, a potential participant will be asked to document her consent by signing an informed consent form. If the participant is a minor (according to the applicable national law), the consent will be signed by a parent or a legal guardian. Nevertheless, an informed assent form must be signed by the minor participant. The signed informed consent (or thumb printed whenever the patient or the parents/guardians are illiterate) must be obtained before any blood sample or tests related to the study are carried out.

#### 2.1.4. Additional Consent Provisions for Collection and Use of Participant Data and Biological Specimens

During the informed consent procedure, study participants (and/or parents/guardians) will be informed about future investigations on the collected blood samples. They will be explained that future investigations will only focus on the understanding of the performance and quality of diagnostic procedures. For this purpose, part of the collected biological specimens may be transported and tested in Amsterdam (The Netherlands). No genetic testing on the human host will be performed.

### 2.2. Interventions

#### 2.2.1. Explanation of the Choice of Comparator

The diagnostic performance of the mini-dbPCR-NALFIA for the screening of malaria and the follow-up of malaria treatment in pregnant women will be compared to the standard malaria diagnostic practices in place in the 2 participating countries (i.e., microscopy and/or rapid diagnostic test). Established qPCR will be used as the reference standard.

#### 2.2.2. Intervention

Both RDT and/or microscopy will be performed as part of the routine diagnostic procedures in place at the study sites. RDT will be performed according to the manufacturer procedures. Thin and thick blood films will be prepared and Giemsa stained. Blood slides will be independently read by 2 expert microscopists. Parasite density will be calculated by counting the number of asexual parasites per 500 leukocytes in the thick blood film, based on an assumed WBC of 8000/µL by light microscopy at 1000× magnification. In addition to routine diagnostic procedures in place, an additional blood sample (preferably finger prick blood) will be collected for test evaluation (index and reference tests). Blood samples will be properly labelled with patients’ initials, inclusion number, protocol number, scheduled study visit, and the date of the sampling. For the index test (mini-dbPCR-NALFIA) <20 μL of blood (collected in an EDTA tube) is needed. For reference testing (qPCR), three blood spots will be collected on Whatman 3 filter paper [31]; one sample for qPCR, the second and third sample for QC procedures (10%) or back-up.

#### 2.2.3. Criteria for Discontinuing or Modifying Allocated Interventions

Only a single additional blood sample will be collected for the screening study. Repeated sampling is needed for the follow-up study but will always involve a single blood sample at the time of follow-up. Treatment decisions will be made on the basis of the PyraPreg study. There will be no interference with standard practices. There is no need to define criteria for discontinuing or modifying the interventions.

#### 2.2.4. Strategies to Improve Adhesion to Intervention

The study is an ancillary study embedded in the PyraPreg trial and has the same sampling points as defined in the PyraPreg trial (day of screening and followed by days 1, 2, 3, 7, 14, 21, 28, 35, 42, 49–56 and 63 for treatment follow-up) and adherence strategy has been developed for PyraPreg trial. Consequently, there is no need to design new strategies to improve adherence to interventions.

#### 2.2.5. Relevant Concomitant Care Permitted or Prohibited during the Trial

Not applicable, as this is a diagnostic study. Prior use of antimalarial drugs might influence diagnostic test performance and then prior drug use in the past 14 days preceding blood collection will be recorded.

#### 2.2.6. Provision for Post-Trial Care

As this is a diagnostic trial in which we are not testing any drug, such visits are not necessary. However, as this is an ancillary study embedded in the PyraPreg trial, post-trial cares have been scheduled until Day 63. These visits have been scheduled physically at the study site. Those who are not recovering well will be managed by the study clinician and recorded in PyraPreg case report forms (CRFs).

#### 2.2.7. Outcomes

The outcome (for the diagnostic screening and treatment follow-up parts) will be reported according to STARD principles [30]. We will report sensitivity, specificity, negative, and positive predictive values (primary outcome) of the mini-dbPCR-NALFIA test. The outcomes of the evaluations will be further analysed using 2 × 2 tables (at 95% confidence intervals), and comparisons of the performance of the diagnostic test under investigation will be made with standard diagnostic procedures in place and qPCR as the accepted reference test to determine the agreement between different tests (expressed as kappa values) [29]. 

To determine the utility of the mini-dbPCR-NALFIA test in monitoring treatment efficacy, parasite clearance will be assessed by mini-dbPCR-NALFIA and compared with qPCR. 

#### 2.2.8. Participant Timeline

Participants will be asked to provide a clinical specimen at the study visit defined by the PryraPreg trial (on the day of screening and days 1, 2, 3, 7, 14, 21, 28, 35, 42, 49–56, and 63 for those who have entered the treatment evaluation).

#### 2.2.9. Sample Size

The sample size for the diagnostic screening component has been calculated according to the expected disease prevalence in the Nanoro health centre catchment area to give adequate statistical power to test sensitivity and specificity [29]. Sample size calculation is based on a 95% confidence interval and using the following equation [29,30]: p +/− 1.96 × √[p(1 − p)/n] where p= sensitivity (or specificity) measured as a proportion and n = number of samples from infected people (or for specificity from non-infected).

The sample size calculation is further based on the estimated prevalence of malaria in pregnancy in the study area, which is around 25% [32]. Based on these assumptions, the following sample size has been determined: 555 cases need to be recruited for the screening component.

For the treatment follow-up component, we will be following the number of cases that are being planned to be recruited for the main PyraPreg trial. In this case, it is expected that we can follow-up with around 138 pregnant women who are under anti-malaria treatment for a period of 63 days.

#### 2.2.10. Recruitment

Prior to the start of the study, communities and community leaders will be informed about the purpose of the study to enhance participation. All study sites have a sufficiently large population and malaria incidence to reach the projected sample size.

### 2.3. Assignment of Intervention: Allocation

#### 2.3.1. Sequence Generation

Not applicable, as there will be no allocation to different study arms. All subjects will be subjected to the same procedures; i.e., collection of a blood sample for diagnostic testing.

#### 2.3.2. Concealment Mechanism

Not applicable, as there will be no allocation to different study arms. All cases will receive the same intervention.

#### 2.3.3. Implementation

Not applicable, as there will be no allocation to different study arms. All cases will receive the same intervention.

### 2.4. Assignment of Interventions: Blinding

There is no procedure for blinding as all participants will be following the same study procedure. 

### 2.5. Data Collection and Management

#### 2.5.1. Plans for Assessment and Collection of Outcomes

Study staff will be trained to perform the study tests in the framework of this trial, and collect and record the study data. All study data will be firstly collected on paper source documents: paper CRFs) and subsequently entered into electronic e-CRFs. For this study, e-CRFs will be developed on Open Clinica software. This can be definite and entered remotely by using the internet or entered offline on a laptop and synchronized with the main database at a later time. The e-CRF will allow identification of the study, site, and patient; recording of the selection and inclusion of patients in the study, and recording of all data collected at each study site.

#### 2.5.2. Plans to Promote Participant Retention and Complete Follow-Up

The study is an ancillary study embedded in the PyraPreg trial and has the same sampling points as defined in the PyraPreg trial (day of screening and days 1, 2, 3, 7, 14, 21, 28, 35, 42, 49–56, and 63 of follow-up) and a plan to promote participant retention or to complete follow-up has been developed in detail for the Pyrapreg trial. Consequently, there is no need to design a new plan to promote participant retention or to complete follow-ups.

#### 2.5.3. Data Management

Project data management will be handled by the Institut de Recherche en Sciences de la Santé (IRSS) which has the required expertise in all components of data management of clinical trials and epidemiological studies. The data management office is located on the main campus of the Clinical Research Unit of Nanoro (CRUN). The CRUN data management currently manages more than 10 ongoing research projects, including clinical trials on treatments, vaccines, and other preventive interventions. Data will be stored at the central server located at CRUN. The central database will be managed by a dedicated data manager, who will run consistency checks and produce queries to be resolved. The final database will be obtained after the resolution of all queries and will be locked for statistical analysis.

In terms of archiving, the Principal Investigator will prepare and maintain a file containing the essential documents for the conduct of the trial. CRF, consent forms of each study subject, and other relevant documents will be archived for 5 years. Direct access to source data and documents relating to the trial will be granted to the sponsor for trial-related monitoring, audits and Ethics Committee review, and regulatory inspections when applicable.

#### 2.5.4. Confidentiality

Every possible measure to ensure that all personal data collected during the study will be appropriately protected will be taken. The collection of personal information will be restricted to meet the objectives of the study, and only data relevant to the execution of the study and interpretation of data will be collected. Collected information will be kept for a minimal time period, which is regulated by national or international law. All personal data collected during the study will be kept confidential. After the collection of blood samples, a numerical identifier will be assigned to each sample. None of the results will be published with individual name identification. At the end of the project, all names will be destroyed. All personalised data forms, signed informed consent forms and other personalised information will be kept under lock and will be available only to the project coordinator, principal investigators, and data managers. Information stored in the electronic database will be protected by unique usernames and passwords, which will be made only available to appropriate authorised personnel. Data entry will be checked independently on site by the research clinician and/or by the PI to ensure accuracy. The collected information will never be transferred to countries lacking appropriate data protection or made available without restriction to third parties that are not directly linked to the project.

Plans for collection, laboratory evaluation, and storage of biological specimens for genetic or molecular analysis in this trial/future use

Blood samples will be collected for molecular analysis by PCR, which is used as a reference test for the diagnostic procedures followed in this trial. This work will be restricted to the assessment of the presence of *Plasmodium* species only. There will be no analysis of human genetic material. Collected specimens could be used for future diagnostic test development and evaluation and explicit informed consent from study participants will be obtained for this.

### 2.6. Statistical Methods

#### 2.6.1. Statistical Methods for Primary and Secondary Outcomes

As mentioned above, the outcomes of the evaluations will be analysed using 2 × 2 tables (at 95% confidence intervals), and comparisons will be made with standard diagnostic procedures in place and qPCR as the accepted reference test. Positive and Negative Predictive Values will be calculated. The sensitivity and specificity of the mini-dbPCR-NALFIA are assumed to be at least 90% [23]. The utility of the mini-dbPCR-NALFIA to monitor treatment success (in terms of recurrent parasitemia) will be compared to qPCR. The association between the molecular test results obtained during the follow-up will be assessed using logistic regression models.

#### 2.6.2. Interim Analysis

Not applicable.

#### 2.6.3. Methods for Additional Analyses (e.g., Subgroup Analyses)

Not applicable.

#### 2.6.4. Methods in Analysis to Handle Protocol Non-Adherence and Any Statistical Methods to Handle Missing Data

The study is an ancillary study embedded in the PyraPreg trial and has the same sampling points as defined in the PyraPreg trial. Only an additional blood sample will be collected at each visit for the test. In case missing data occur during a visit, the whole data set of a participant at this visit will be removed from the analysis. This removal will be compensated by a slight over recruitment in the number of study participants. Nevertheless, a missing visit will not affect the data analysis of other visits of the participant.

#### 2.6.5. Plans to Give Access to the Full Protocol, Participant Level-Data and Statistical Code

The study has been registered in an accessible clinical trial register (PACTR202203780981413) and the information contained in that register provides sufficient insight into the full trial design. The final results of the study will be published in peer-reviewed Open Access journals and presented at scientific meetings. None of the trial material may be disclosed to any party not directly involved in the study without written permission from the project partners. Presentation and publication of the trial results will be jointly carried out by the project investigators. Each African collaborator will be in charge of sharing relevant results with their respective health authorities, including the National Malaria Control Programmes. This will be done through ad-hoc meetings, data dissemination workshops, and/or during the celebration of African Malaria Day in each country. Trial data will be accessible for inspection by appropriate health and regulatory authorities.

### 2.7. Oversight and Monitoring

#### 2.7.1. Composition of the Coordinating Center and Trial Steering Committee

The trial is coordinated by a dedicated trial staff based at CRUN (Nanoro, Burkina Faso). CRUN is responsible for generic study protocol development and implementation. Each country is responsible for the ethical review of the study protocol and the implementation of the study at its respective site.

#### 2.7.2. Composition of the Data Monitoring Committee, Its Role and Reporting Structure

CRUN is responsible for the centralized data management and monitoring. The central database will be managed at CRUN by a dedicated data manager, who will run regular consistency checks and produce queries to be resolved by the local investigators. This system will allow the rapid identification of potential problems. Data entry and review will be performed following the Data Management Plan. Data management is independent of the sponsor.

#### 2.7.3. Adverse Event Reporting and Harms

The intervention is unlikely to result in (unexpected) adverse events or harm. This is an ancillary study embedded in the PyraPreg project and the incidental findings policy will follow the one set-up for the PyraPreg project. This study will not involve research conducted on genes and/or the genetic basis of diseases in the human subject. The research is not intended to provide subjects or their families with specific information about their genetic status. Furthermore, the research will be focused on the diagnostic of malaria only (a very common and well-known condition in performance study) and not on any other diseases or conditions and does not actively search for human genetic disorders. Therefore, any other medical problem detected in patients with malaria or those tested negative for these infections will be sent to the nearest health facility for further medical management. The risk that incidental findings occur in this study is very low though it might be the case when blood slides are examined by microscopy, and there is a chance that other parasites, like Trypanosomes or bloodstream microfilariae, will be found. In such a case, the attending physician will be informed to undertake appropriate medical actions.

#### 2.7.4. Frequency and Plans for Auditing Trial Conduct

Each site will be visited by an independent monitor at least once during the conduct of the trial plus a study initiation visit at the start of the study activities and a close-out visit after the last patient has completed the follow up. The task of the Monitor is to verify the best conduct of the study through frequent contacts by phone and in person with the Principal Investigator and site staff, in accordance with applicable regulations, Good Clinical Practice requirements, and study-specific Standard Operating Procedures. The objectives and specific tasks of the Monitor are described in the ICH Guidelines E6. Source documents will be kept for each patient in the study. All information in the electronic CRF must be traceable to these source documents in the patient’s file. The monitor, who is bound by a confidentiality agreement to protect patients’ confidentiality, has access to all relevant source documents to confirm their consistency with the electronic CRF entries.

#### 2.7.5. Plans for Communicating Important Protocol Amendments to Relevant Parties (e.g., Trial Participants, Ethical Committees)

Protocol amendments will be communicated to the sponsor (AMC) and relevant ethical review bodies in the country concerned.

#### 2.7.6. Dissemination Plans

Dissemination of the study findings is planned through scientific papers and conference attendance, and also through meetings with various audiences of interest, including key stakeholders such as public health authorities and diagnostic companies who might be interested in bringing the diagnostic platform to market.

## 3. Discussion

Pregnant women require specific consideration in malaria control programs in endemic countries. Indeed, the increasing susceptibility to malaria infection during pregnancy, makes pregnant women living in endemic areas, an important reservoir of *Plasmodium* in the community [33]. The high susceptibility of pregnant women in many endemic countries interferes with diagnostic testing during pregnancy, rending target interventions ineffective for control [34]. This situation becomes exacerbated when available diagnostics have a low detection limit [6,7,8,9,10] and are unable to detect low peripheral parasitemia due to the sequestration of the parasite in the placenta [35]. Although microscopy is still considered to be the reference test for the diagnostic of malaria, despite the limit of detection [17], it is unfortunately not readily available in rural areas for reasons such as the lack of qualified staff and sufficient laboratory infrastructure. This is also the case for molecular biology for which specific equipment and highly trained technicians are needed in order to be adequately performed [19]. In order to propose an alternative diagnostic tool for the correct diagnosis of malaria in endemic areas particularly in pregnant women and where suitable laboratory facilities are lacking, an innovative diagnostic platform combining direct blood amplification in a miniaturized PCR system with NALFIA as a read-out to diagnose *Plasmodium* infections in a multiplex format has been developed.

The present trial has been designed to assess the diagnostic performance of this platform for the detection of *Plasmodium* species in pregnant women during antenatal consultation and treatment follow-up. The proposed trial will validate the anticipated low limit of detection and sensitivity and specificity of the new diagnostic platform. If these diagnostic parameters are achieved, this study will provide the evidence needed for the introduction of the mini-dbPCR-NALFIA in the routine health system for a correct diagnosis of malaria and treatment follow-up during pregnancy.

**Trial status:** This is the protocol Version 2.0, of 11 March 2021. Ethical approvals have been obtained for both participating countries. At the time of this manuscript submission, study participants’ enrollment has started in Burkina Faso (8 November 2021), and in DRC (7 December 2021). The trial is ongoing with an anticipated completion date in December 2023.

## Data Availability

Final trial outcomes will be published in Open Access peer reviewed journals. Anonymized datasets generated and analyzed during this study will be available for up to 5 years after completion of the project upon a reasonable motivated request to the corresponding author.

## Abbreviations

ACT	Artemisinin Combination Therapy
AMC	Academic Medical Centre
CRF	Case Report Form
CRUN	Clinical Research Unit of Nanoro
DRC	Demographic Republic of Congo
dbPCR-NALFIA	direct-on-blood PCR
DNA	Deoxyribonucleic Acid
eCRF	Electronic Case Report Form
EDCTP	European & Developing Countries Clinical Trials Partnership
EDTA	ethylenediaminetetraacetic acid
HRP2	histidine-rich protein 2
HIV	Human Immunodeficiency Virus
IRSS	Institut de Recherche en Sciences de la Santé
LoD	Limit of detection
NALFIA	Nucleic Acid Lateral Flow Immunoassay
[n]PoC	near Point-of-Care
*P. falciparum*	*Plasmodium falciparum*
PCR	polymerase chain reaction
*Pf*HRP2	*Plasmodium falciparum* histidine-rich protein 2
*p*LDH	*Plasmodium* lactate dehydrogenase
PoC	Point-of-care
QC	Quality Control
qPCR	quantitative PCR
RDTs	Rapid Diagnostic Test
STARD	Standards for Reporting of Diagnostic Accuracy Studies
TDR	Special Programme for Research and Training in Tropical Diseases
UV	ultra violet
WBC	white blood cells
WHO	World Health Organization
